# Pathologically confirmed spontaneous regression of small cell lung cancer after computed tomography-guided percutaneous transthoracic needle biopsy followed by surgery

**DOI:** 10.1186/s40792-023-01759-9

**Published:** 2023-10-25

**Authors:** Madoka Goto, Koichi Fukumoto, Yasuhisa Ichikawa, Hideki Tsubouchi, Mika Uchiyama, Shoichi Mori

**Affiliations:** Department of Thoracic Surgery, Japanese Red Cross Aichi Medical Center Nagoya Daiichi Hospital, 3-35 Michishita-Cho, Nakamura-Ku, Nagoya, 453-8511 Japan

**Keywords:** Primary lung cancer, Spontaneous regression, Small cell lung cancer, Computed tomography-guided percutaneous transthoracic needle biopsy (CT-guided PTNB), Pulmonary resection, Lobectomy, Immune response

## Abstract

**Background:**

Spontaneous regression of malignant tumors is a rare phenomenon, especially in primary lung cancer. The underlying mechanisms remain unclear, but they may often involve immunological mechanisms.

**Case presentation:**

In January 2020, a 78-year-old female underwent examination during follow-up of interstitial pneumonia. Chest X-ray and computed tomography (CT) scan revealed a 1.2 × 1.2 cm nodule in the left lower lobe. Based on CT-guided percutaneous transthoracic needle biopsy (PTNB), it was diagnosed as small cell lung cancer (SCLC). Immunohistochemical staining showed that tumor cells were positive for CD56, synaptophysin, and chromogranin A. Twenty-three days after the CT-guided PTNB, repeat CT scan showed that the tumor size regressed to 0.6 × 0.6 cm. The tumor showed positive uptake in fluorodeoxyglucose (FDG) positron emission tomography (PET)–CT. The maximum standardized uptake value of the nodule was 2.24. PET–CT and enhanced magnetic resonance imaging of the brain showed no distant or lymph node metastasis. The patient’s preoperative disease was diagnosed as cT1aN0M0, stageIA1, SCLC. In March 2020, she underwent left lower lobectomy and mediastinal lymph node dissection. Pathological examination of the resected specimen showed that the small tumor cells were dense with a high nucleus to cytoplasm ratio, and the morphological diagnosis was small cell carcinoma. The resected tumor size regressed to 0.05 × 0.02 cm, and no lymph node metastasis was observed. Because it was extremely small, immunohistochemical staining could not be conducted. Active fibrosis and inflammation were present around the tumor. Finally, the patient was pathologically diagnosed as SCLC pT1miN0M0, stage IA1. The patient is alive without recurrence 23 months after surgery with no adjuvant therapy.

**Conclusions:**

We present a rare surgical case of pathologically confirmed spontaneous regression of SCLC after CT-guided PTNB. Although spontaneous regression is extremely rare, we should recognize this phenomenon.

## Background

Spontaneous regression (SR) is an extremely rare phenomenon, where malignant tumors either partially or completely disappearance without appropriate treatment. Cole et al. reported that the incidence of SR in malignant tumors is 1 per 60,000–100,000 cases [1], with many cases reported in lymphoma, hepatic cancer, and neuroblastoma; it is rarely seen in primary lung cancer [2, 3]. Its underlying mechanisms are unclear but often involve immunological mechanisms. Herein, we report a case of small cell lung cancer (SCLC) that spontaneously regressed in size after computed tomography-guided percutaneous transthoracic needle biopsy (CT-guided PTNB).

## Case presentation

**Fig. 1:**
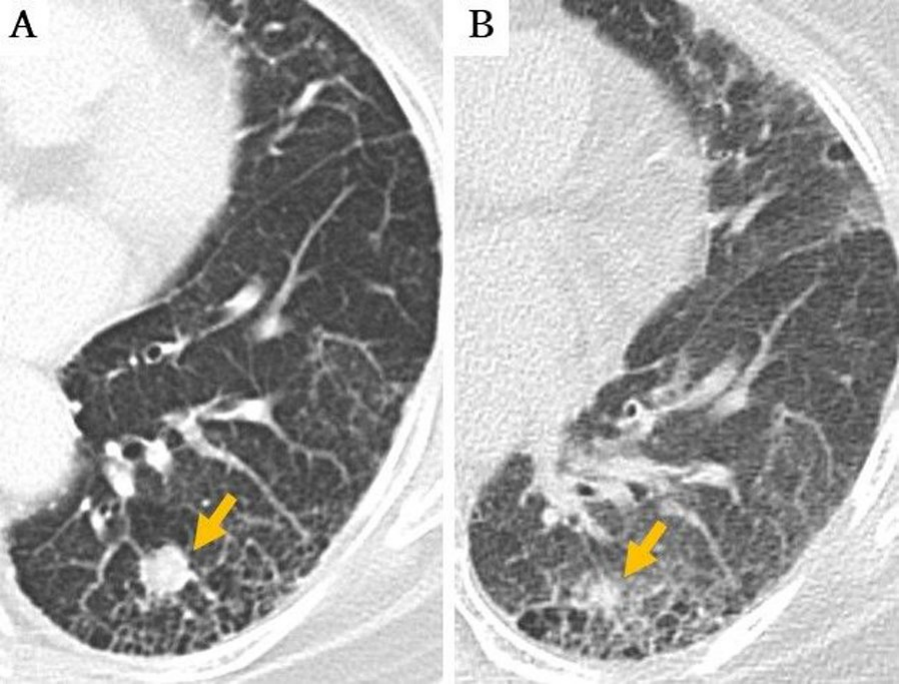
Chest computed tomography (CT) before and after CT-guided percutaneous transthoracic needle biopsy (CT-guided PTNB). **A** In February 2020, chest CT revealed a 1.2 × 1.2 cm solid nodule in the posterior segment of the left lower lobe. **B** In March 2020, follow-up CT revealed that the nodule regressed in size from 1.2 × 1.2 cm to 0.6 × 0.6 cm

**Fig. 2:**
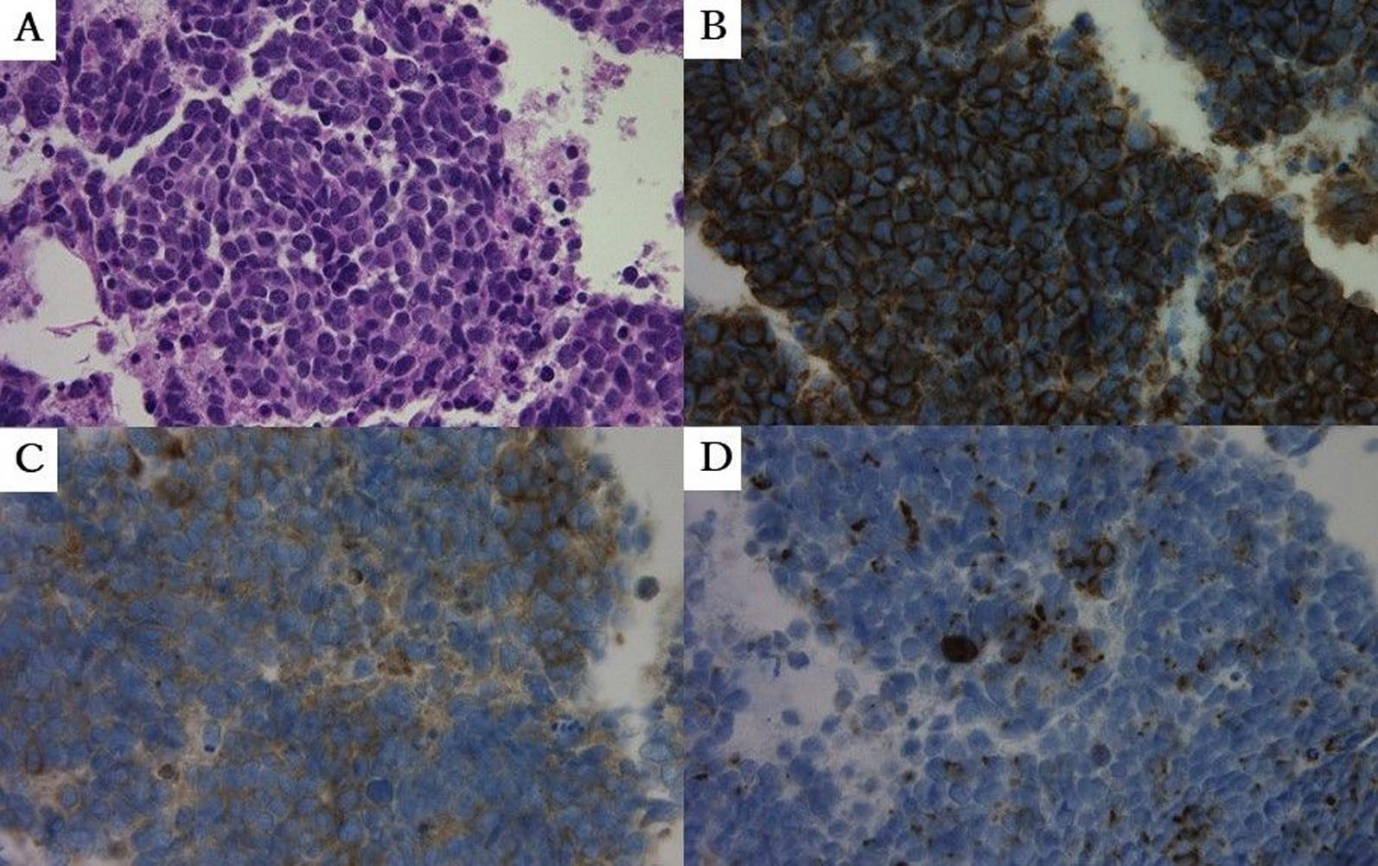
Pathological findings of the computed tomography-guided percutaneous transthoracic needle biopsy (CT-guided PTNB) specimen. Pathological examination of CT-guided PTNB specimens revealed dense small cells with a high nucleus-to-cytoplasm ratio (**A**, HE × 200). Immunohistochemical staining showed that the tumor cells were positive for CD56 (**B**, HE × 200), synaptophysin (**C**, HE × 200), and chromogranin (**D**, HE × 200)

A 78-year-old female with primary lung cancer in the left lower lobe (LLL) was referred to our hospital for surgical intervention. She had a history of interstitial pneumonia (IP), tuberculosis, and hypothyroidism. She was neither taking any medication nor any herbal remedies or supplements. During a follow-up for IP, chest computed tomography (CT) revealed a pure solid nodule measuring 1.2 cm in maximal diameter in the posterior segment of LLL (Fig. 1A). There were also basal-dominant reticular patterns and basal and subpleural honeycomb-like lesions, which are a usual IP (UIP) pattern. Blood examination showed that the levels of tumor markers, such as carcinoembryonic antigen, cytokeratin fragment, and progastrin-releasing peptide, were all within normal ranges. Serum Krebs von den Lungen-6 antigen level was elevated (1014 U/mL). Pathological examination of CT-guided PTNB specimens revealed dense small cells with high nucleus to cytoplasm (N/C) ratio (Fig. 2A). An 18-gauge, 11-cm long needle was used for the biopsy, which was performed for one time. Immunohistochemical staining showed that the tumor cells were positive for CD56 (Fig. 2B), synaptophysin (Fig. 2C), and chromogranin A (Fig. 2D). The nodule was pathologically diagnosed as SCLC.

The nodule in LLL showed a positive uptake in fluorodeoxyglucose (FDG) positron emission tomography (PET)–CT; the maximum standardized uptake value was 2.24. Repeat chest CT images 23 days after the CT-guided PTNB revealed an ill-defined nodule that regressed in size (from 1.2 × 1.2 cm to 0.6 × 0.6 cm: Fig. 1B). No metastatic lesions were detected by enhanced brain magnetic resonance imaging or abdominal CT and the patient was diagnosed with cT1aN0M0, stageIA1, and SCLC. No exacerbation of interstitial pneumonia was observed.

**Fig. 3:**
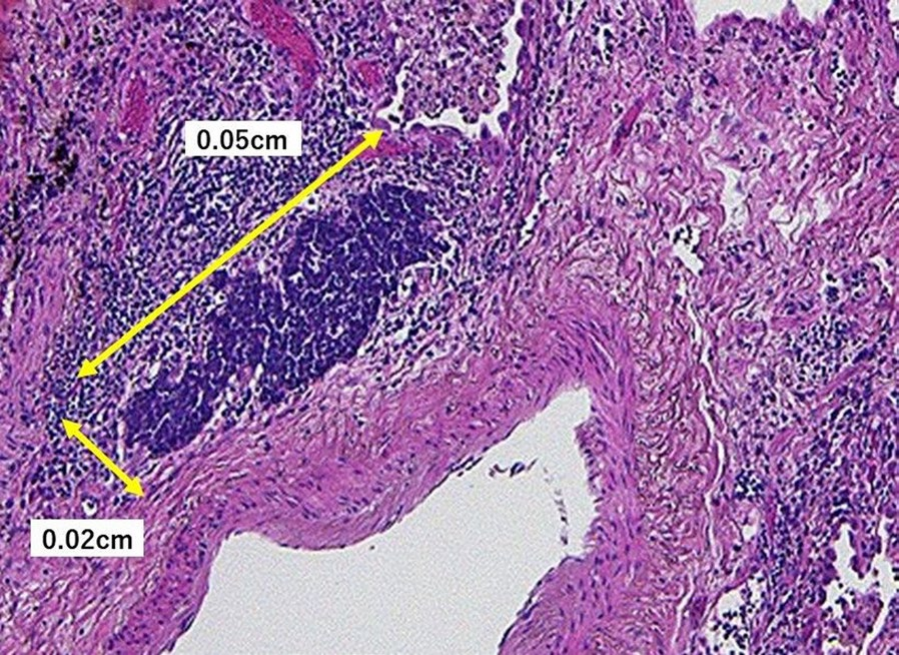
Pathological findings of the resected specimens (HE stain, high-power field). Pathological examination showed a tiny lung cancer lesion (0.05 × 0.02 cm). Due to its size, immunohistochemical staining was impossible

**Fig. 4:**
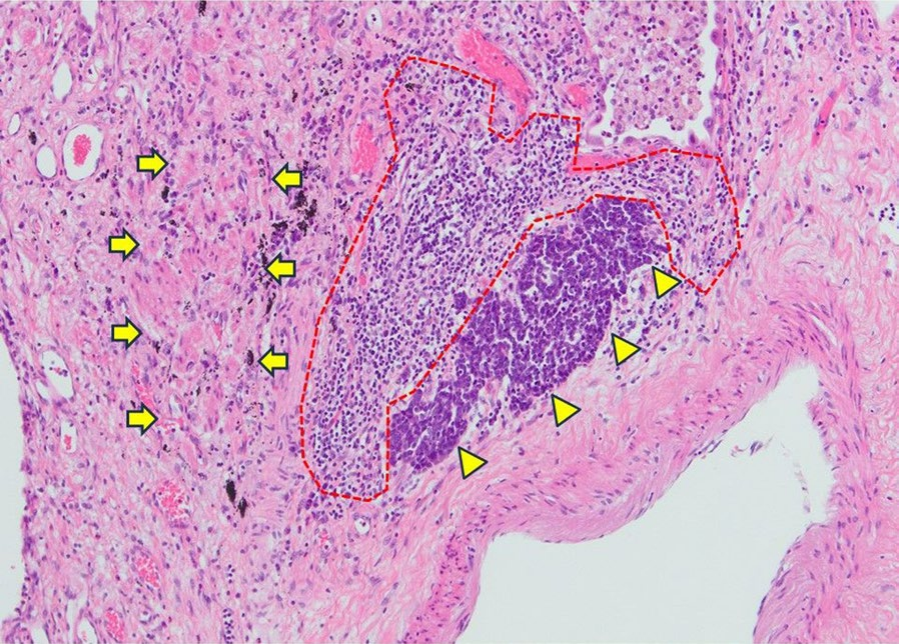
Pathological findings of the resected specimens (HE stain, low-power field). Pathological findings revealed an active fibrosis (arrows) and lymphocyte infiltration (dotted line) around the tiny lung cancer lesion (arrowheads)

**Fig. 5:**
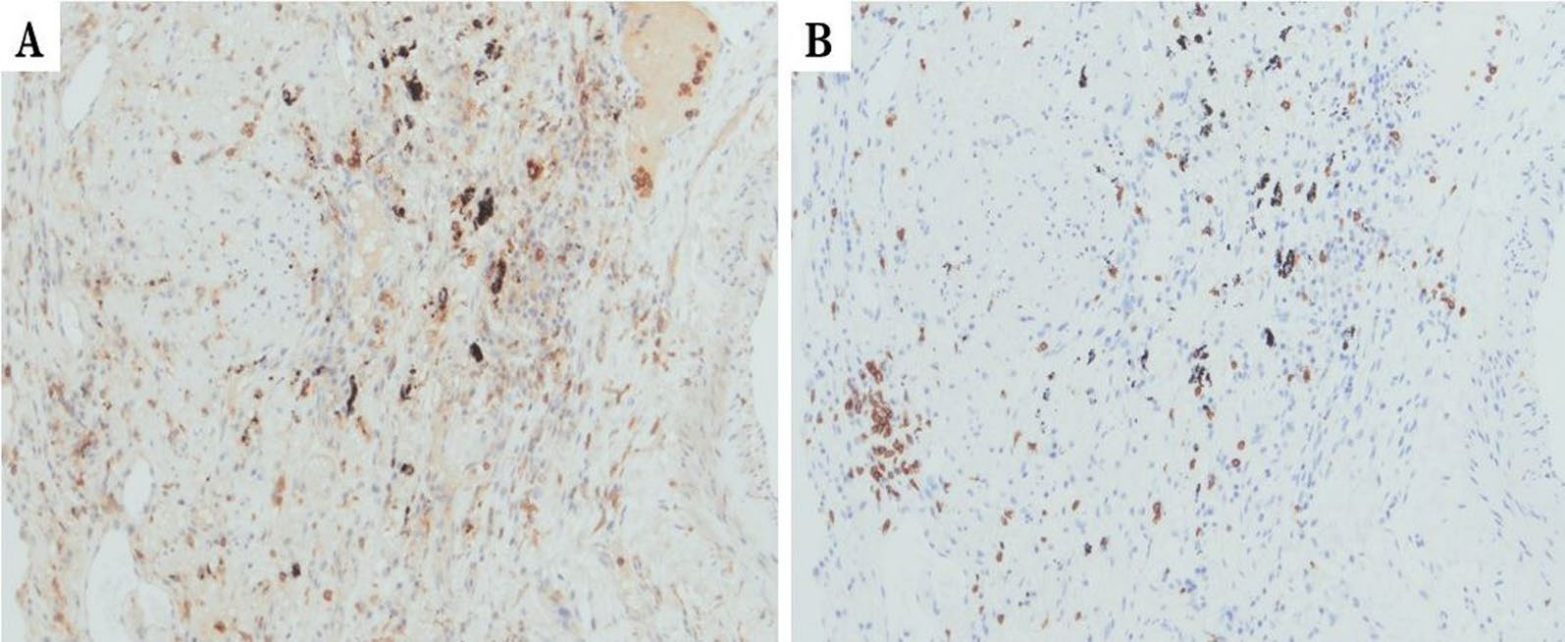
Immunohistochemical staining of the resected specimens. CD4-positive (**A**) and CD-8 positive (**B**) T-lymphocytes were observed around the tumor. The immunohistochemical staining of the tumor could not be conducted due to its extremely small size

In March 2020 (29 days after the CT-guided PTNB), left lower lobectomy with mediastinal lymph node dissection was performed. The tumor could not be identified macroscopically. Pathological examination showed a tiny lung cancer lesion (0.05 × 0.02 cm) (Fig. 3). Due to its extremely small tumor size, immunohistochemical staining for chromogranin A or synaptophysin could not be conducted. It was dense with a high N/C ratio, which resembled the SCLC in CT-guided PTNB specimens. The final morphological diagnosis was small cell carcinoma. Active fibrosis and infiltration of CD4/8-positive T-lymphocytes were observed around 1 cm of the tumor, with no necrosis or vessel embolization (Figs. 4, 5). No lymph node metastasis was observed. Finally, the patient was pathologically diagnosed with pT1miN0M0 stage IA1 SCLC with UIP. The patient was discharged on the 10th postoperative day without any complications. The patient received no adjuvant therapy because of old age and the presence of UIP. The patient is alive without recurrence of lung cancer 23 months after surgery.

## Discussion

SR of malignant tumors is a rare phenomenon defined as the partial or complete disappearance of the tumor without any appropriate treatment [1]. SR of malignant tumors is commonly reported in neuroblastoma, renal cell cancer, lymphoma, and hepatic cancer [3, 4]. SR among thoracic malignancies is most frequent in primary lung cancer [4], although few cases of SR of thymic epithelial tumors were also reported [5, 6]. In terms of the histological type of lung cancer with SR, Iwanaga et al. reported that adenocarcinoma accounted for 45%, followed by squamous cell carcinoma (20%) and SCLC (20%) 4.

A literature review of SR in SCLC, including our case, is shown in Table 1 [7–16]. The median age was 70 years (ranging from 55 to 83 years), with three males and eight females. SR was pathologically proven only in this study. Of the nine patients whose prognostic information was available, three patients survived for more than 5 years, and notably two patients survived for more than 10 years. In general, the prognosis of patients with SCLC is poorer than that of patients with non-SCLC, with a median survival rate of 2–4 months when not treated [17, 18]. The 5-year survival rate of SCLC is reported to be below 7% [19]. Given that six patients in Table 1 had no treatment and only two underwent surgical resection, the prognosis of SCLC with SR cases might be better than that of SCLC without SR. Although the tumor has completely regressed, such as other histologic subtypes, a careful attention to metastasis or regrowth should still be given [20]. A long-term follow-up also is warranted.

**Table 1:** Spontaneous regression (SR) of small cell lung cancer: a review of literature

	First author	Years	Age	Sex	Biopsy before SR	Shrinkage percentage (duration)	Treatment modality after SR	Stage	OS (months)
1	Shibata^7^	2021	83	F	TBNA	100% (7 years)	BSC	Unknown	108
2	Song^8^	2021	80	M	Bronchoscopic biopsy	Unknown (52 months)	BSC	Unknown (LD)	53
3	Ugajin^9^	2019	82	M	TBLB	100% (18 months)	BSC	c-IIIB	18
4	Inui^10^	2015	69	F	TBLB	37.5% (unknown)	Surgery (segmentectomy) Adjuvant chemotherapy (CDDP + ETP)	p-IA	27
5	Kitai^11^	2014	65	F	EBUS–TBNA	Unknown (unknown)	Chemotherapy (CDDP + ETP)	c-IIIB	Unknown
6	Iwakami^12^	2013	56	F	VATS biopsy	Partial regression (30 days)	CRT (CDDP + ETP and RT)	c-IIIB	25
7	Mawhinney^13^	2010	Elderly	F	biopsy	Partial regression (7 months)	BSC	Unknown	18
8	Lee^14^	2008	70	F	Bronchoscopic biopsy	100% (unknown)	BSC	c-IIIA	132
9	Hirano^15^	2007	55	F	TBLB	Partial regression (1 month)	CRT (CDDP + ETP and RT)	c-IIIA	Unknown
10	Lowy^16^	1986	55	M	Node biopsy	Unknown (6 months)	BSC	Unknown	228
11	Our case		78	F	CT-guided PTNB	95.8% (1 month)	Surgery (lobectomy)	p-IA1	23

*M* male, *F* female, *EBUS–TBNA* endobroncial ultrasound-guided transbronchial needle aspiration, *TBLB* transbronchial lung biopsy, *PTNB* percutaneous transthoracic needle biopsy, *LD* limited disease, *PNS* paraneoplastic syndrome, *BSC* best supportive care, *RT* radiation therapy, *UIP* usual interstitial pneumonia, *CRT* chemoradiotherapy, *CDDP* cisplatin, *ETP* etoposide, *OS* overall survival

SR of small cell carcinoma was reported in the esophagus, bronchus, and parotid gland [21–23]. In all cases, SR was observed after a diagnostic biopsy. The mechanism of SR is unclear, but factors that stimulate an immune response, including infection, trauma, surgery, and blood transfusions, may be involved [1]. In this case, active fibrosis, inflammation, and lymphocyte infiltration were observed around the regressed tumor. These findings implied that the SR in this case was caused by some immunological response after the CT-guided PTNB conducted 29 days before surgery. The lymphocytes around the tumor were CD4/8 positive T cells, which play an important role in cancer cytotoxicity. Previous reports have suggested that cytotoxicity by T cells may be associated with SR [12]. The cell-mediated immunity induced by PTNB may be a possible cause of SR in this case. We are unsure why this small residual tumor existed on this site; however, it can be speculated that the fibrotic lesion was associated with a decreased blood flow. Although some cases have radiologically confirmed partial or complete SR, this is the first and rare case that histologically verified SR, with inflammation and infiltration of lymphocytes around the tumor. Analyzing its underlying mechanisms may lead to more effective treatment for cancer.

## Conclusions

We present a surgical case of pathologically confirmed SR of SCLC after CT-guided PTNB. This is an extremely rare case that pathologically verified not only SR but also inflammation and infiltration of lymphocytes around the tumor. Although SR is rare, we should recognize this phenomenon.

## Data Availability

Data sharing does not apply in this article as no data sets were generated or analyzed during the current study.

## Abbreviations

CT: Computed tomography
PTNB: Percutaneous transthoracic needle biopsy
SCLC: Small cell lung cancer
FDG: Fluorodeoxyglucose
PET: Positron emission tomography
SR: Spontaneous regression
LLL: Left lower lobe
IP: Interstitial pneumonia
UIP: Usual interstitial pneumonia
